# Impact of *Clostridium difficile* infection among pneumonia and urinary tract infection hospitalizations: an analysis of the Nationwide Inpatient Sample

**DOI:** 10.1186/s12879-015-0925-9

**Published:** 2015-07-01

**Authors:** Monideepa B. Becerra, Benjamin J. Becerra, Jim E. Banta, Nasia Safdar

**Affiliations:** Department of Health Science and Human Ecology, California State University, San Bernardino, USA; William S Middleton Memorial Veterans Hospital, Madison, Wisconsin USA; Primary Address: 5500 University Parkway, San Bernardino, CA 92407 USA; School of Public Health, Loma Linda University, Primary Address: 24951 North Circle Drive, Loma Linda, CA 92350 USA; University of Wisconsin, Primary Address: 1685 Highland Ave., Madison, Wisconsin 53705 USA

**Keywords:** *Clostridium difficile*, Urinary tract infection, Pneumonia, Nationwide inpatient sample, Hospital acquired infection, Nosocomial infection

## Abstract

**Background:**

*Clostridium difficile* infection (CDI) remains one of the major hospital acquired infections in the nation, often attributable to increased antibiotic use. Little research, however, exists on the prevalence and impact of CDI on patient and hospital outcomes among populations requiring such treatment. As such, the goal of this study was to examine the prevalence, risk factors, and impact of CDI among pneumonia and urinary tract infection (UTI) hospitalizations.

**Methods:**

The Nationwide Inpatient Sample (2009–2011), reflecting a 20% stratified sample of community hospitals in the United States, was used. A total of 593,038 pneumonia and 255,770 UTI discharges were included. Survey-weighted multivariable regression analyses were conducted to assess the predictors and impact of CDI among pneumonia and UTI discharges.

**Results:**

A significantly higher prevalence of CDI was present among men with UTI (13.3 per 1,000) as compared to women (11.3 per 1,000). CDI was associated with higher in-hospital mortality among discharges for pneumonia (adjusted odds ratio [aOR] for men = 3.2, women aOR = 2.8) and UTI (aOR for men = 4.1, women aOR = 3.4). Length of stay among pneumonia and UTI discharges were also double upon presence of CDI. In addition, CDI increased the total charges by at least 75% and 55% among pneumonia and UTI discharges, respectively. Patient and hospital characteristics associated with CDI included being 65 years or older, Charlson Deyo index for comorbidity of 2 or more, Medicare as the primary payer, and discharge from urban hospitals, among both pneumonia and UTI discharges.

**Conclusion:**

CDI occurs frequently in hospitalizations among those discharged from hospital for pneumonia and UTI, and is associated with increased in-hospital mortality and health resource utilization. Interventions to mitigate the burden of CDI in these high-risk populations are urgently needed.

## Background

*Clostridium difficile* infection (CDI) is a leading cause of hospital-acquired infection (HAI) [1-3]. In many areas of the United States, CDI has surpassed methicillin-resistant *Staphylococcus aureus* as the most common type of HAI [1] with approximately 333,000 initial and 145,000 recurrent hospital-onset cases in the nation [4]. Certain patient populations have a disproportionately higher risk for CDI due to either host factors, frequent antibiotic use or both. These include older adults, patients using proton pump inhibitors [5-7] or antibiotics [8,9], those with inflammatory bowel disease [10-12], end-stage renal failure, or recipients of solid organ transplants [13,14].

In a study addressing the burden of CDI among patients with inflammatory bowel disease, Ananthakrishnan et al. [12] demonstrated four times higher mortality and three days longer hospital stay with presence of CDI. Similarly, among solid organ transplant patients, presence of CDI significantly increased the in-hospital mortality, length of stay, and charges, in addition to organ complications [14]. Despite such recognized burden of CDI, limited research exists on the prevalence and impact of infection among most common conditions that require antimicrobial treatment, with no study to date evaluating such impact among pneumonia or urinary tract infection (UTI) patients. Some recent empirical evidence has noted the co-occurrence of pneumonia and UTI with CDI in the United States [15] putatively due to the use of antimicrobial treatment; though none have evaluated the impact of such co-occurrences on patient and hospital outcomes. Misdiagnosis of pneumonia and inappropriate use of antimicrobial therapy was associated with a CDI outbreak [16]. Given the burden of CDI nationally and increasing prevalence attributed at least in part to antibiotic use, understanding the impact of CDI among patients with pneumonia or UTI would be valuable to devise potential preventive strategies. We undertook analyses of an existing large dataset from a nationally representative survey to assess [1] the prevalence and factors associated with CDI among pneumonia and UTI and [2] the impact of CDI on in-hospital mortality and health resource utilization (length of stay [LOS] and total charges).

## Methods

### Data source

Data was extracted from the Nationwide Inpatient Sample (NIS), 2009–2011. NIS, considered the largest publically available all-payer inpatient database in the United States, includes data from all states that participate in the Healthcare Cost and Utilization Project (HCUP), sponsored by the Agency for Healthcare Research and Quality (AHRQ). An annual approximate sample of 8 million hospitalizations from 1,000 hospitals, reflecting a 20% stratified sample of community hospitals in the nation are included in NIS. NIS excludes short-term rehabilitation hospitals (starting 1998 data), long-term non-acute care hospitals, psychiatric hospitals, and alcoholism/chemical dependency treatment facilities. All hospitals in NIS are stratified based on five hospital characteristics: ownership/control, bed size, teaching status, urban/rural location, and geographic location. Starting 1988, NIS data is available yearly and further details of the dataset are available elsewhere [17].

### Data collection and study definitions

Our study sample included hospital primary discharges with pneumonia or UTI in adults over the age of 17 years. The International Classification of Diseases, 9^th^ Revision, Clinical Modification (ICD-9-CM) was used to identify UTI (599.0) and CDI (008.45). Pneumonia was assessed using Clinical Classification Software (CCS) code of 112, representing cases not caused by tuberculosis or sexually transmitted disease (as defined by NIS). Similar coding strategies have been utilized in previous literature for CDI [11,13,14], pneumonia [18,19], and UTI [20,21]. NIS provides a primary discharge code in addition to additional codes of secondary diagnoses. In this study, patients with secondary discharge code for CDI were identified as CDI patients.

We further included both patient and hospital characteristics, to both control for and evaluate if such characteristics negatively impact CDI patients. For example, previous research has demonstrated both patient socioeconomic factors and hospital characteristics, to be positively associated with length of stay and mortality in other patient populations [11,13,14,22,23], and thus such variables were further accounted for in our study.

Patient characteristics included were: age (18–34 years, 35–49 years, 50–64 years, 65 years or more), gender (men, women), race/ethnicity (White, Black, Hispanic, Asian or Pacific Islander, Native American, other), primary payer type (Private including HMO, Medicare, Medicaid, Self-pay, no charge, other), neighborhood income defined as median household income quartiles by patient ZIP code ($1-$38999, $39000-$47999, $48000-$62999, $63000 or more) and Charlson-Deyo Index (0, 1, 2 or more). The Deyo modification of the Charlson comorbidity index was used, which creates a score representing co-morbidities for each discharge utilizing the ICD-9-CM coding algorithms. The 17-item index is a validated measure of comorbidity for administrative data [24-26].

Hospital characteristics included in the study were: bed size tertile categories (small, medium, large), ownership/control (private investor-own, private non-profit, government nonfederal), setting/teaching status (rural, urban non-teaching, urban teaching), and geographic location (Northeast, Midwest, South, West). In-hospital mortality was defined as those who died during hospitalization versus those who did not. LOS and total charges were used from NIS-provided variables, which were edited by AHRQ to ensure uniformity between states. Total charges were adjusted quarterly for inflation using the Gross Domestic Product (GDP) deflator available through the United States Department of Commerce, Bureau of Economic Analysis with 2009 USD as the reference year [27].

### Statistical analyses

SAS 9.4 (SAS Institute, Inc., Cary, NC) was used for all statistical analyses except for negative binomial regression, for which we used the STATA 12 package (Stata Corp LP, College Station, TX). Given that existing data suggests potential gender differences in CDI [28-30] all statistical analyses were stratified by gender. Due to the large number of variables and in turn multiple testing, a family-wise correction using the Bonferroni adjustment was conducted to reduce type I error rate. As a result, *P* < 0.0017 was set as the level of significance.

To assess CDI prevalence (for each patient population), in addition to patient and hospital characteristic differences between each gender among pneumonia and UTI cases, chi-square tests using design-based *F* values were used. The prevalence of secondary CDI was noted as cases per 1,000 discharges for pneumonia and UTI groups, by gender. Next, independent survey-weighted multivariable logistic regression analyses were performed to identify patient and hospital characteristics associated with prevalence of secondary CDI in both primary pneumonia and UTI discharges.

In order to identify the impact of secondary CDI on in-hospital mortality among patients hospitalized for primary pneumonia or UTI, chi-square tests were conducted followed by survey-weighted logistic regression analyses. To assess the impact of secondary CDI on resource utilization, Wilcoxon rank sum test was used, followed by survey-weighted negative binomial regression and survey-weighted linear regression for LOS and total charges, respectively. For all adjusted models in each regression analyses, control variables of survey year, patient, and hospital characteristics were included. Since the distribution of total charges was non-normal and skewed to the right, this variable was natural log transformed for linear regression analyses. In addition, given that descriptive analyses demonstrated a significantly higher percent of our population as 65 or older and the elderly are more likely to have negative health impacts [31-33], a sensitivity analysis was performed in the aforementioned adjusted models among patients aged 65 and older. Model building for all analyses included assessment of assumptions and relevant interaction terms (sociodemographic characteristics with hospital characteristics), with significance established at *P* < .05. The study was submitted to the University of Wisconsin-Madison Institutional Review Board and was considered exempt from review.

## Results

A total of 593,038 pneumonia and 255,770 UTI discharges were included in this study. Of them, 6,427 cases of secondary CDI among pneumonia patients and 3,037 secondary CDI cases among UTI patients were noted, representing 10.8 secondary CDI cases per 1,000 pneumonia discharges and 11.1 secondary CDI cases per 1,000 UTI discharges.

Gender specific analyses found a total of 2,996 and 1,000 cases of secondary CDI among men with pneumonia and UTI, respectively. Among women, 3,431 cases of secondary CDI were noted among those hospitalized for pneumonia and an additional 2,037 cases among those with primary UTI. Table 1 summarizes the prevalence of secondary CDI, patient, and hospital characteristics among primary pneumonia and UTI discharges, by gender. While rates of CDI among those with pneumonia did not differ between each gender, significant difference was noted for UTI patients, with men reporting 13.3 cases of secondary CDI per 1,000 compared to 11.3 cases per 1,000 for women (*P* < 0.001). As further noted in Table 1, several patient and hospital characteristics were significantly different among men and women and as a result all such variables were included in final model building for regression analyses.

**Table 1:** **Prevalence of CDI, patient and hospital characteristics among primary pneumonia and UTI discharges, NIS 2009-2011**

	Pneumonia			UTI		
	Men	Women	*P* value	Men	Women	*P* value
n	279,072	313,966		75,600	180,170	
N	462,171	519,849		125,338	298,156	
**Prevalence of secondary CDI, cases per 1,000**	10.7	10.9	0.52	13.3	11.3	<0.001
**Patient Characteristics**						
**Age, %**						
18-34 years old	5.7	5.0		3.2	3.8	
35-49 years old	10.6	10.4	<0.001	6.3	5.4	<0.001
50-64 years old	22.4	21.2		15.7	11.8	
65 years old or more	61.3	63.3		74.9	78.9	
**Race/ethnicity, %**						
White	75.4	75.5		71.8	74.9	
Black	11.5	12.0		14.9	12.1	
Hispanic	7.8	7.5	<0.001	8.7	8.3	<0.001
Asian or Pacific Islander	2.0	1.8		1.5	1. 7	
Native American	0.8	0.8		0.6	0.6	
Other	2.6	2.4		2.6	2.3	
**Charlson-Deyo Index, %**						
0	20.8	21.3		28.0	33.0	
1	26.7	31.8	<0.001	23.5	27.3	<0.001
2 or more	52.5	46.8		48.5	39.8	
**Neighborhood income, %**						
$1 - $38,999	32.0	32.9		30.1	30.4	
$39,000 - $47,999	27. 2	27.0	<0.001	24.9	25.4	0.009
$48,000 - 62,999	23.0	22.8		24.0	23.9	
$63,000 or more	17.7	17.3		21.0	20.3	
**Payer type, %**						
Private including HMO	19.1	17.9		12.2	10.4	
Medicare	64.5	66.7		77.2	79.6	
Medicaid	8.4	9.8	<0.001	6.8	6.7	<0.001
Self-pay	4.8	3.6		1.8	2.1	
No Charge	0.5	0.4		0.2	0.2	
Other	2.6	1.7		1.7	1.0	
**Hospital Characteristics**						
**Bed size, %**						
Small	19.0	19.7		16.4	16.8	
Medium	24.9	25.2	<0.001	24.8	25.3	0.06
Large	56.1	55.2		58.8	57.9	
**Hospital control, %**						
Private, investor-own	14.4	14.7		15.3	16.2	
Private, non-profit	71.0	71.3	0.001	70.8	71.3	<0.001
Government, nonfederal	14.6	14.0		13.9	12. 6	
**Setting, %**						
Rural	22.1	22.7		16.6	17.8	
Urban non-teaching	44.0	44.8	<0.001	44.9	46.3	<0.001
Urban teaching	33.9	32.5		38.4	35.8	
**Region, %**						
Northeast	18.8	18.0		22.1	19.9	
Midwest	25.3	25.6	<0.001	22.5	22.7	<0.001
South	38.9	40.6		40.4	42.7	
West	16.9	15.9		15.1	14.7	
**Year, %**						
2009	34.1	34.1		32.5	32.0	
2010	32.3	32.1	0.24	34.0	33.3	0.005
2011	33.5	33.8		33.5	34.7	

CDI = *Clostridium difficile* infection, UTI = Urinary tract infection, n = total sample size, N = weighted average annual population estimate, CI = confidence interval

Table 2 displays the factors significantly associated with secondary CDI among primary pneumonia and UTI discharges. Among hospitalizations for pneumonia, increased odds of CDI were associated with being 65 years or older (adjusted odds ratio [aOR] for men = 1.7; aOR for women = 1.9), having Medicare as the primary payer (aOR men and women = 1.3), and increasing Charlson Deyo index (aOR men = 1.5; aOR women = 1.8). CDI was also significantly associated with high income (aOR = 1.3) and Medicaid (aOR = 1.4) among men hospitalized for pneumonia. Furthermore, for both men and women, hospitalization at urban non-teaching facilities was associated with approximately 60% increased odds of CDI while nearly double the odds were noted if hospitalized at urban teaching centers. On the other hand, lower odds were noted among men admitted at government nonfederal hospitals, as compared to investor-owned facilities (aOR = 0.7).

**Table 2:** **Determinants of CDI among pneumonia and UTI discharges, NIS 2009-2011**

	Pneumonia		UTI	
	Men	Women	Men	Women
Patient characteristics				
**Age**				
18-34 years old (ref.)				
35-49 years old	0.98 (0.74, 1.31)	0.84 (0.61, 1.14)	0.82 (0.49, 1.38)	1.49 (0.96, 2.33)
50-64 years old	1.27 (0.97, 1.65)	1.34 (1.00, 1.78)	0.84 (0.53, 1.33)	1.92 (1.19, 3.11)
65 years old or more	1.70 (1.28, 2.25)*	1.85 (1.40, 2.45)*	0.94 (0.61, 1.44)	2.41 (1.47, 3.95)*
**Race/ethnicity**				
White (ref.)				
Black	0.92 (0.79, 1.07)	1.02 (0.89, 1.16)	1.08 (0.87, 1.34)	1.01 (0.86, 1.18)
Hispanic	1.02 (0.86, 1.21)	0.84 (0.71, 1.00)	0.85 (0.63, 1.15)	0.88 (0.73, 1.08)
Asian/Pacific Islander	1.26 (0.97, 1.62)	0.91 (0.69, 1.20)	0.96 (0.55, 1.67)	0.88 (0.60, 1.28)
Native American	1.50 (0.86, 2.62)	0.84 (0.50, 1.43)	0.57 (0.14, 2.31)	0.66 (0.28, 1.56)
Other	1.14 (0.87, 1.50)	1.14 (0.87, 1.47)	1.24 (0.82, 1.87)	0.84 (0.58, 1.21)
**Charlson-Deyo Index**				
0 (ref.)				
1	0.98 (0.86, 1.13)	1.16 (1.02, 1.32)	0.88 (0.72, 1.09)	1.18 (1.03, 1.36)
2 or more	1.54 (1.36, 1.75)*	1.81 (1.59, 2.05)*	1.31 (1.10, 1.55)*	1.72 (1.53, 1.94)*
**Neighborhood income**				
$1 - $38,999 (ref.)				
$39,000 - $47,999	1.08 (0.96, 1.23)	1.07 (0.95, 1.21)	1.09 (0.86, 1.37)	0.89 (0.77, 1.03)
$48,000 - 62,999	1.12 (0.98, 1.27)	1.10 (0.97, 1.25)	1.29 (1.04, 1.59)	1.17 (1.01, 1.36)
$63,000 or more	1.30 (1.13, 1.49)*	1.23 (1.07, 1.43)	1.52 (1.21, 1.90)*	1.17 (0.98, 1.39)
**Payer type**				
Private including HMO (ref.)				
Medicare	1.32 (1.15, 1.51)*	1.26 (1.09, 1.45)*	1.21 (0.95, 1.55)	0.90 (0.74, 1.10)
Medicaid	1.38 (1.14, 1.68)*	1.19 (0.98, 1.45)	1.04 (0.72, 1.50)	1.00 (0.78, 1.28)
Self-pay	0.69 (0.49, 0.96)	0.87 (0.61, 1.23)	0.25 (0.08, 0.78)	0.53 (0.30, 0.94)
No Charge	1.28 (0.66, 2.50)	0.54 (0.17, 1.72)	0.74 (0.13, 4.42)	0.38 (0.05, 2.75)
Other	1.10 (0.81, 1.49)	1.08 (0.70, 1.67)	0.86 (0.44, 1.67)	0.51 (0.26, 1.03)
**Hospital characteristics**				
**Bed size**				
Small (ref.)				
Medium	0.92 (0.78, 1.09)	1.03 (0.88, 1.20)	1.02 (0.80, 1.30)	0.88 (0.74, 1.04)
Large	1.09 (0.94, 1.27)	1.17 (1.01, 1.35)	1.19 (0.96, 1.48)	0.97 (0.83, 1.14)
**Hospital control**				
Private investor-own (ref.)				
Private non-profit	0.79 (0.68, 0.92)	0.86 (0.74, 1.00)	1.01 (0.79, 1.28)	1.15 (0.96, 1.37)
Government nonfederal	0.68 (0.55, 0.84)*	0.76 (0.63, 0.93)	0.88 (0.63, 1.23)	1.06 (0.84, 1.35)
**Setting**				
Rural (ref.)				
Urban non-teaching	1.63 (1.36, 1.96)*	1.66 (1.40, 1.98)*	1.63 (1.20, 2.20)	1.42 (1.16, 1.73)*
Urban teaching	2.05 (1.71, 2.47)*	1.92 (1.60, 2.30)*	1.92 (1.42, 2.61)*	1.74 (1.42, 2.14)*
**Region**				
Northeast (ref.)				
Midwest	0.94 (0.79, 1.11)	0.87 (0.73, 1.04)	0.99 (0.79, 1.23)	1.04 (0.86, 1.25)
South	0.79 (0.68, 0.91)	0.78 (0.66, 0.92)	0.82 (0.66, 1.01)	0.81 (0.68, 0.96)
West	0.83 (0.70, 0.97)	0.82 (0.69, 0.98)	0.78 (0.61, 1.01)	0.87 (0.72, 1.05)
**Year**				
2009 (ref.)				
2010	1.10 (0.97, 1.24)	0.96 (0.85, 1.08)	1.11 (0.93, 1.32)	1.03 (0.90, 1.19)
2011	1.06 (0.94, 1.20)	0.95 (0.84, 1.06)	1.06 (0.89, 1.26)	1.01 (0.88, 1.16)

CDI = *Clostridium difficile* infection, UTI = Urinary tract infection

*Bonferroni adjusted *P* < 0.0017

Similar trends were noted for UTI discharges. Being 65 years or older (aOR = 2.4 for men only), highest income category (aOR = 1.5 for men only), increasing comorbidities (aOR men = 1.3; aOR women = 1.7), urban teaching status (aOR men = 1.9; aOR women = 1.7), and urban non-teaching status (aOR = 1.4 for women only) were also significantly associated with increased likelihood of secondary CDI.

Results of chi-square analyses demonstrated that in-hospital mortality was higher among pneumonia patients diagnosed with CDI, as compared to those without, for both men (13% vs. 4%, *P* < 0.0001) and women (11% vs. 4%, *P* < 0.0001). A similar effect of CDI among UTI discharges was noted in regards to in-hospital mortality among men (4% vs. 1%, *P* < 0.0001) and women (3% vs. 1%, *P* < 0.0001).

Table 3 shows the results of regression analyses (unadjusted and adjusted) to evaluate the impact of CDI on in-hospital mortality and resource utilization among pneumonia and UTI cases. After adjusting for control variables, in-hospital mortality was approximately three times higher for pneumonia cases with CDI as compared to those without CDI. Having CDI among men hospitalized for UTI also increased the likelihood of in-hospital mortality by four times. Similarly, among women with UTI, CDI was associated with three times the odds of dying in the hospital.

**Table 3:** **Regression analyses of in-hospital mortality and health resource utilization upon secondary CDI among patients with pneumonia or urinary tract infection** ^**a**^

	In-hospital mortality		Health resource utilization			
	OR (95% CI)		Length of stay		Total charge	
			IRR (95% CI)		% change (95% CI)	
	Unadjusted	Adjusted^b^	Unadjusted	Adjusted^b^	Unadjusted	Adjusted^b^
Primary pneumonia with secondary CDI^c^						
Women	3.42	2.84	2.40	2.29	86.14	74.9
	(3.03, 3.86)*	(2.51, 3.21)*	(2.30, 2.50)*	(2.19, 2.39)*	(80.36, 91.92)*	(70.66, 79.20)*
Men	3.70	3.15	2.54	2.40	93.65	80.00
	(3.28, 4.17)*	(2.79, 3.55)*	(2.43, 2.66)*	(2.30, 2.52)*	(87.93, 99.38)*	(75.56, 84.48)*
Primary UTI with secondary CDI^d^						
Women	3.69	3.39	2.19	2.11	62.93	56.92
	(2.82, 4.84)*	(2.58, 4.44)*	(2.06, 2.32)*	(1.99, 2.24)*	(57.08, 68.78)*	(52.41, 61.44)*
Men	4.05	4.13	2.14	2.13	67.37	59.34
	(2.89, 5.67)*	(2.95, 5.78)*	(1.99, 2.29)*	(1.99, 2.29)*	(59.23, 75.50)*	(52.30, 66.38)*

*Bonferroni adjusted *P* < 0.0017

CDI = *Clostridium difficile* infection, UTI = Urinary tract infection, OR = odds ratio, CI = confidence interval, IRR = incidence rate ratio

^a^Binary logistic regression for in-hospital mortality, negative binomial regression for length of stay, linear regression for total charges. All procedures were survey weighted

^b^Model adjusted for patient characteristics, hospital characteristics, and year

^c^Reference = primary pneumonia no secondary CDI

^d^Reference = UTI no secondary CDI

Wilcoxon rank sum tests demonstrated that median LOS was significantly longer in pneumonia cases with CDI as compared to those without CDI for both men and women (9 days vs. 4 days, *P* < 0.0001). Similar trends were noted for UTI with or without CDI (7 days vs. 3 days, *P* < 0.0001). Adjusted results of negative binomial regression analyses showed that having CDI for pneumonia and UTI discharges was associated with approximately 200% increased LOS (Table 3).

Among men with pneumonia, higher median total charges ($104131 vs. $41157, *P* < 0.0001) were noted upon presence of CDI, with a similar trend reported among women ($96446 vs. $40700, *P* < 0.0001). Median total charges were also substantially higher upon UTI cases with CDI for both men ($63842 vs. $34182, *P* < 0.0001) and women ($33063 vs. $61577, *P* < 0.0001) as well. Results from multiple linear regression analyses showed that secondary CDI was significantly associated with increased percent change in total charges for both pneumonia and UTI cases. For example, presence of secondary CDI increased total charges by 80% and 75% among men and women with pneumonia, respectively. Similarly, CDI was associated with 59% and 57% increase in total charges among men and women with UTI, respectively (Table 3). After conducting a sensitivity analysis among ages 65 and older, a similar trend persisted for in-hospital mortality, LOS, and total charges (Table 4). Cumulatively, presence of secondary CDI among pneumonia or UTI discharges was substantially associated with increased in-hospital mortality and health resource utilization.

**Table 4:** **Regression analyses of in-hospital mortality and health resource utilization upon secondary CDI among patients ages 65+ with pneumonia or urinary tract infection** ^**a**^

	In-hospital mortality	Health resource utilization	
		Length of stay	Total charge
	OR (95% CI)^b^	IRR (95% CI)^b^	% change (95% CI)^b^
Primary pneumonia with secondary CDI^c^			
Women	2.67 (2.34, 3.05)*	2.18 (2.08, 2.29)*	71.04 (66.4, 75.73)*
Men	3.06 (2.69, 3.48)*	2.25 (2.14, 2.35)*	74.52 (69.76, 79.27)*
Primary UTI with secondary CDI^d^			
Women	3.59 (2.73, 4.71)*	2.06 (1.94, 2.18)*	54.99 (50.20, 59.78)*
Men	4.35 (3.07, 6.16)*	2.07 (1.93, 2.23)*	57.13 (49.17, 65.08)*

*Bonferroni adjusted *P* < 0.0017

CDI = *Clostridium difficile* infection, UTI = Urinary tract infection, OR = odds ratio, CI = confidence interval, IRR = incidence rate ratio

^a^Binary logistic regression for in-hospital mortality, negative binomial regression for length of stay, linear regression for total charges. All procedures were survey weighted

^b^Model adjusted for patient characteristics, hospital characteristics, and year

^c^Reference = primary pneumonia no secondary CDI

^d^Reference = UTI no secondary CDI

## Discussion

Our study found that CDI is highly prevalent in patients with pneumonia or UTI and is associated with significantly increased in-hospital mortality, LOS, and total charges. To our knowledge, this study is the first of its kind to examine secondary CDI using a nationally representative dataset in patients with pneumonia or UTI, both frequent conditions that often require hospitalization and antimicrobial therapy.

In our analyses, the prevalence of CDI is considerably higher compared to that reported among general hospitalized patients, but lower than prevalence noted among those with conditions that may uniquely predispose patients to CDI, such as ulcerative colitis [11,12]. We also found a gender difference in CDI prevalence, with higher rates noted among men with UTI as compared to women. Such gender differences may be attributable to differences in antibiotic prescribing practices as duration of treatment for men with UTI is generally longer [34-37]. Future prospective studies should further corroborate these findings and explore underlying mechanisms.

A major finding in our study was that LOS was doubled for both men and women with secondary CDI as compared to those without. Moreover, at least 75% and 55% increases in total charges were noted among pneumonia and UTI patients with CDI, respectively. In-hospital mortality was also substantially higher among patients in secondary CDI as compared to those without. Our findings on the negative impact of CDI on patient and hospital outcomes are in line with results noted among other patient populations with CDI, such as inflammatory bowel disease, solid organ transplant, and end stage renal failure [11-14]; reflecting the substantial burden of CDI in vulnerable patients.

We identified a number of hospital and patient characteristics associated with CDI in patients with primary pneumonia or UTI. Consistent with our results, previous studies have reported a greater risk of CDI with increasing age [29,38]. A study assessing *C. difficile* colitis case fatality rate also noted higher rates among those in Medicare and Medicaid [30], demonstrating the negative burden of CDI among patients with such insurance status, similar to trends noticed in our findings. We hypothesize that given that Medicare patients are elderly, they may be at a greater risk of negative outcomes and thus the result noted in our study. In addition, Medicaid has traditionally been for low income populations, and thus limited resources, budget constraints, restrictions, etc. could contribute to the negative rates. Not surprisingly and in keeping with other studies, we found that increasing number of comorbidities, as reflected by the Charlson Deyo index, was associated with a greater risk for CDI [30].

We also reported higher odds of secondary CDI among urban hospitals. Possible reasons include greater complexity of illness or higher antibiotic prescribing practices among urban hospitals [39], though some researchers have illustrated the opposite [40] or non-significant differences between urban or rural settings [41]. Future studies to examine this issue with additional ways to account for case mix are needed. Our findings of worse outcomes in this large swath of hospitalized patients suggest opportunities for both infection prevention and stewardship in this population.

Our findings have major implications for clinicians and healthcare institutions and add to the body of literature on the prevalence and impact of CDI. The substantial negative impact of secondary CDI on in-hospital mortality and health resource utilization emphasizes that those hospitalized for pneumonia or UTI represent high-risk patient populations for CDI and should be included in CDI preventive efforts. Opportunities for optimizing antimicrobial therapy use among patients to reduce such burden exists, such as severity-based treatment, de-escalation as appropriate, and adherence to treatment guidelines for choice and duration of anti-infectives [42,43]. Future studies to examine interventions for reducing the risk of and mitigating the consequences of CDI in patients with pneumonia and UTI are urgently needed.

Our results should be interpreted in the context of study limitations. Given that NIS does not provide patient identifiable information, such ICD-9-CM codes could not be cross-validated with laboratory results. Previous research, however, has shown the validity and use of such codes for CDI detection [44,45]. Second, the unit of observation in NIS is discharges and not individual patients; therefore results of this study cannot assess the impact of initial versus recurrent CDI. Third, while patient level characteristics, such as age, gender, race/ethnicity, payer type, and neighborhood income based on zip codes are included in the NIS dataset, other social or medical determinants, such as health literacy, education, dietary factors and treatments, including outpatient procedures, could not be evaluated.

## Conclusion

In our study, using the largest inpatient data in the United States, we demonstrated that men have a significantly higher prevalence of CDI, as compared to women. CDI was also associated with increased in-hospital mortality for both pneumonia and UTI patients, as well as increased LOS and total charges; further highlighting the negative impact of CDI and imperative need for preventive measures.

## Abbreviations

CDI: *Clostridium difficile* infection
LOS: Length of stay
NIS: Nationwide Inpatient Sample
UTI: Urinary tract infection
